# Surface expression of the immunotherapeutic target G_D2_ in osteosarcoma depends on cell confluency

**DOI:** 10.1002/cnr2.1394

**Published:** 2021-04-02

**Authors:** Malena Wiebel, Sareetha Kailayangiri, Bianca Altvater, Jutta Meltzer, Kay Grobe, Sabine Kupich, Claudia Rossig

**Affiliations:** ^1^ Department of Pediatric Hematology and Oncology University Children's Hospital Muenster Muenster Germany; ^2^ Institute of Physiological Chemistry and Pathobiochemistry University of Muenster Muenster Germany; ^3^ Cells‐in‐Motion Cluster of Excellence (EXC 1003 ‐ CiM) University of Muenster Muenster Germany

**Keywords:** cellular immunotherapy, chimeric antigen receptors, gangliosides, G_D2_, osteosarcoma

## Abstract

**Background:**

Chimeric antigen receptor (CAR) T‐cell therapy of pediatric sarcomas is challenged by the paucity of targetable cell surface antigens. A candidate target in osteosarcoma (OS) is the ganglioside G_D2_, but heterogeneous expression of G_D2_ limits its value.

**Aim:**

We aimed to identify mechanisms that upregulate G_D2_ target expression in OS.

**Methods and results:**

G_D2_ surface expression in OS cells, studied by flow cytometry, was found to vary both among and within individual OS cell lines. Pharmacological approaches, including inhibition of the histone methyltransferase Enhancer of Zeste Homolog 2 (EZH2) and modulation of the protein kinase C, failed to increase G_D2_ expression. Instead, cell confluency was found to be associated with higher G_D2_ expression levels both in monolayer cultures and in tumor spheroids. The sensitivity of OS cells to targeting by G_D2_‐specific CAR T cells was compared in an in vitro cytotoxicity assay. Higher cell confluencies enhanced the sensitivity of OS cells to G_D2_‐antigen specific, CAR T‐cell‐mediated in vitro cytolysis. Mechanistic studies revealed that confluency‐dependent upregulation of G_D2_ expression in OS cells is mediated by increased *de novo* biosynthesis, through a yet unknown mechanism.

**Conclusion:**

Expression of G_D2_ in OS cell lines is highly variable and associated with increasing cell confluency in vitro. Strategies for selective upregulation of GD2 are needed to enable effective therapeutic targeting of this antigen in OS.

**Abbreviations**
5′UTR: 5′ untranslated region
BFA: Brefeldin A
CAR: chimeric antigen receptor
CAT: chloramphenicol acetyltransferase
CD: cluster of differentiation
DMSO: dimethyl sulfoxide
DNA: deoxyribonucleic acid
EZH2: enhancer of Zeste Homolog 2
G_D2_
: ganglioside D2
GD2S: GD2 synthase
GD3S: GD3 synthase
GFP: green fluorescent protein
HER2: human epidermal growth factor receptor 2
hrs: hours
IC_30_
: 30% inhibitory concentration
IFN‐γ: interferon γ
IgG: immunoglobulin G
IRES: internal ribosomal entry site
mAb: monoclonal antibody
mRNA: messenger ribonucleic acid
OS: osteosarcoma
PKC: protein kinase C
PMA: phorbol‐12‐myristat‐13‐acetat
RFI: relative fluorescence intensity

## 1 INTRODUCTION

Osteosarcoma (OS) is the most common primary malignancy of bone in children and adolescents. Adjuvant chemotherapy combined with surgical resection is the key to successful treatment.^1^ Attempts by international cooperative groups to intensify cytotoxic regimens have not succeeded in further improving outcomes since the 1970s.^2^, ^3^ The addition of a biological agent, liposomal muramyl tripeptide phosphatidyl ethanolamine (L‐MTP‐PE), yielded an increase of survival in patients with non‐metastatic disease,^3^ but had no benefit in metastatic OS.^4^ Recurrences typically occur in the lungs, with dismal outcome despite repeated surgery.^5^ Novel therapeutic approaches are needed to eliminate (micro)metastatic disease and prevent relapse.

Cellular immunotherapy with chimeric antigen receptor (CAR) engineered T cells has shown striking efficacy against refractory B‐cell cancers.^6^, ^7^ Whereas the limited clinical consequences of on‐target depletion of normal B cells allow to target B lineage markers, solid tumors lack surface antigens exclusively expressed on tumor cells and not on indispensable normal cells. A candidate in OS is the disialoganglioside antigen G_D2_. G_D2_ is abundantly expressed on immature neuroectodermal tissues during embryogenesis, whereas postnatal expression is low and restricted to neuronal and mesenchymal stromal cells (reviewed in Reference 8). G_D2_ was found to be a safe therapeutic target for antibodies and CAR T cells in neuroblastoma, where it is abundantly expressed.^9^, ^10^ Immunohistochemistry studies have found aberrant expression of G_D2_ also in proportions of patients with Ewing sarcoma^11^ and OS,^12^, ^13^, ^14^ with preserved expression at recurrence.^15^ But in contrast to neuroblastoma, G_D2_ expression in sarcomas is heterogeneous among patients and within individual tumors. To avoid antigen‐negative escape, G_D2_‐specific immunotherapy in these cancers will have to be combined with strategies that upregulate target expression to homogeneous levels.

In previous studies in Ewing sarcoma, our group has shown that inhibitors of the histone methyltransferase Enhancer of Zeste Homolog 2 (EZH2) upregulate G_D2_ expression, associated with the reversal of silencing of genes encoding for enzymes in G_D2_ biosynthesis, effectively sensitizing antigen‐negative/low tumor cells to G_D2_‐targeted cell therapy.^16^ Overexpression of EZH2 was reported also in OS where it is associated with a highly aggressive tumor phenotype and poorer prognosis.^17^, ^18^ Here, we investigated strategies to upregulate G_D2_ also in OS, starting with the hypothesis that epigenetic modification by inhibition of EZH2 could induce GD2 expression also in this cancer.

## 2 METHODS

### 2.1 Cell lines

All OS cell lines were purchased from ATCC and the early passages after receiving were expanded and frozen in batches. The identity of the cell lines was confirmed by short tandem repeat (STR) profiling directly before freezing and the cells used for the experiments were cultured for a maximum of six passages after thawing. Tumor cells were cultured in uncoated tissue culture flasks in RPMI 1640 medium (Invitrogen, Germany) supplemented with 10% heat‐inactivated fetal calf serum (FCS; Thermo Scientific, Waltham, Massachusetts) and 2 mM l‐glutamine (Sigma‐Aldrich, St. Louis, Missouri) at 37°C and 5% CO_2_. One hundred U/mL penicillin and 100 μg/mL streptomycin (Thermo Scientific, Waltham, Massachusetts) were added during long‐term assays. The medium was changed every 3 to 4 days. Adherent sarcoma cells were harvested by trypsinization. The assays were performed by experienced individuals throughout the course of the study. The study was performed using established laboratory protocols covering the processing, freezing, storage, and thawing of cells as well as the staining procedure, data acquisition, and gating strategy. Raw data can be provided per request.

### 2.2 Flow cytometry analysis

For the analysis of G_D2_ expression, 100,000 tumor cells were stained with phycoerythrin (PE)‐conjugated monoclonal antibody (mAb) against G_D2_ (14.G2a) or the corresponding PE‐labeled isotype anti‐IgG2a (both BioLegend, Germany). Dead cells were excluded from analysis by additional staining with Zombie Violet (Bio‐Legend, Germany). Samples were fixed with 1% paraformaldehyde (PFA) and acquired directly or not later than 24 hours after staining. For each sample, 10,000 cells within the respective gates were analyzed with FACS Diva 8.0 using FACS Celesta flow cytometer (BD Biosciences, Germany) and FlowJo version 10 (FlowJo, USA). Relative fluorescence intensities (RFI) were calculated by dividing median fluorescence intensities of mAb‐stained cells by those obtained with isotype antibodies (IgG2a): RFI=median_GD2_/median_isotype_.

### 2.3 Treatment with EZH2 inhibitor

OS cells were harvested and seeded in uncoated six‐well plates (Sarstedt, Germany) at 0.1 to 0.5 × 10^6^ cells/well in a total volume of 2 mL. After 2 hours, the EZH2 inhibitor tazemetostat (Cayman Chemicals, Ann Arbor, Michigan) dissolved in DMSO or DMSO alone as control was added at a concentration of 1, 12, 30 (Saos‐2) or 60 μM (HOS), respectively. After 3 to 4 days of incubation at 37°C and 5% CO_2_, the medium was changed and the EZH2 inhibitor was added again at the same concentration. Every 7 days, the cells were harvested and analyzed for G_D2_ expression as above.

### 2.4 Tumor spheroids

Boiled‐up 1% agarose at 45 μL/well were pipetted into the wells of a flat‐bottom 96‐well plate (Thermo Scientific, Waltham, Massachusetts). After solidification, low‐confluent MG‐63 cells were harvested from monolayer cultures and 5000 cells were seeded to each agarose‐coated well in a volume of 150 μL medium. The plates were incubated at 37°C and 5% CO_2_ and 100 μL of fresh medium was added on day 4. Spheroids were carefully harvested using truncated pipette tips and pooled, then trypsinated and filtrated through a cell strainer (Corning, USA). Finally, cells were stained and analyzed for G_D2_ expression.

### 2.5 Treatment with brefeldin A

The OS cell lines U‐2 OS and MG‐63 were incubated at 37°C and 5% CO_2_. Twenty‐four hrs before reaching 50% or 100% confluency, respectively, 5 mg/mL brefeldin A (BFA) (Sigma‐Aldrich, Germany) or DMSO as control were added. After 24 hours of incubation, cells at 50% or 100% confluency were harvested and analyzed for G_D2_ expression by flow cytometry.

### 2.6 Treatment with protein kinase C modulators

U‐2 OS and MG‐63 cells at low confluencies were incubated with the protein kinase C (PKC) modulators phorbol‐12‐myristat‐13‐acetat (PMA; Sigma‐Aldrich, Germany) at 200 nM for 2, 4, and 12 hours or bryostatin 1 (Sigma‐Aldrich, USA) at 1 ng/mL for 24, 48, and 72 hours at 37°C and 5% CO_2_. Equivalent volumes of DMSO were used as control. After stimulation with PMA or bryostatin 1, cells were harvested and G_D2_ expression was assessed by flow cytometry.

### 2.7 Quantitative real‐time PCR

RNA was isolated using the RNeasy‐Kit (QIAGEN, Germany) according to the manufacturers' instructions. RNA concentration and purity were determined by Nanodrop analysis (Thermo Scientific, Germany). cDNA was synthesized with the NEB protocol (New England Bio‐Labs, Ipswich, Massachusetts). PCR reactions were set up with 1 μL cDNA, 5 μL NEB Luna Universal qPCR Master Mix, 0.5 μL primers, and 3.5 μL H_2_O. Primers for GD3 synthase (GD3S; ST8SIA1, QT00054159; QIAGEN), GD2 synthase (GD2S; B4GALNT1, QT02564009; QIAGEN), and the reference gene HPRT1 (forward primer 5′‐TGAGGATTTGGAAAGGGTGT‐3′, reverse primer 5′‐GAGCACACAGAGGGCTACAA‐3′; Thermo Scientific, Germany) were used. Amplification was performed in triplicate reactions in two different runs at 95°C for 15 minutes, followed by 94°C for 15 seconds and 40 cycles of 55°C (30 s) and 72°C (30 s) on a CFX96 Thermal Cycler (BioRad). Cq values were determined using CFX Manager (BioRad) and adjusted to Cq values of HPRT1 control gene to ensure equal amplification efficiencies. The triplicates of each run were averaged for analysis. Relative gene expression levels were calculated by using the Delta Ct with the formula 2^Ct(HPRT01)‐Ct(GD3S or GD2S)^.

### 2.8 Generation of 5′UTR‐GD3S constructs, transfection and quantification of G_D2_ , GFP, and CAT

The GD3S 5′UTR sequence was purchased from Thermo Scientific (Germany) and cloned into pCAT‐EGFP (pCAT‐5′UTR‐GFP), as previously described.^19^ In detail, the 5′UTR sequence was placed between the chloramphenicol acetyltransferase (CAT) and green fluorescent protein (GFP) coding sequences. Native pCAT‐GFP was used as control. MG‐63 cells were transfected in 10 cm cell culture dishes (Corning, USA) with 10 μg DNA (pCAT‐5′‐GFP or pCAT‐GFP) using XtremeGENE^TM^ HP DNA Transfection Reagent (Sigma‐Aldrich, USA). After 10 hours, cells were harvested and reseeded in six‐well plates at counts of 2 × 10^5^/well or 2 × 10^6^/well, respectively, to establish low‐ and high‐confluent cell cultures. After 38 hours of incubation, cells were harvested and GFP expression was assessed by flow cytometry.

### 2.9 CAR constructs and transduction of human T cells

The CAR gene GD2‐BBζ and the production of recombinant retrovirus for transduction of T cells were previously described.^11^, ^20^, ^21^ Expansion and transduction of T cells from peripheral blood were performed as described.^21^

### 2.10 Cytotoxicity assay

Target cells were stained with 10 μM calcein‐AM (Thermo Scientific, Germany) for 30 minutes. Then, 1 × 10^4^ cells per well were placed in a 96‐well flat‐bottom microtiter plate with CAR T cells at effector‐to‐target cell ratios of 40:1 to 10:1 or alone (spontaneous release). After 4 hours, the supernatant was transferred into black‐walled 96‐well microtiter plates (Greiner Bio‐One, Germany) and fluorescence was quantified with a microplate reader GloMax Discover (Promega, Germany). To determine maximum release, cells were lysed with 9% Triton X‐100. Data were expressed as Arbitrary Fluorescence Units (AFU) and specific lysis was calculated by [(test release − spontaneous release)/(maximum release − spontaneous release)] × 100.

### 2.11 Statistical analysis

Data were analyzed and visualized using SigmaPlot 11.0 software (Systat Software, USA). Statistical analysis was performed as indicated in the figure legends. Results were considered statistically significant at *P* ≤ .05.

## 3 RESULTS

### 3.1 EZH2 inhibition fails to upregulate G_D2_ surface expression in OS cells to high levels

**FIGURE 1:**
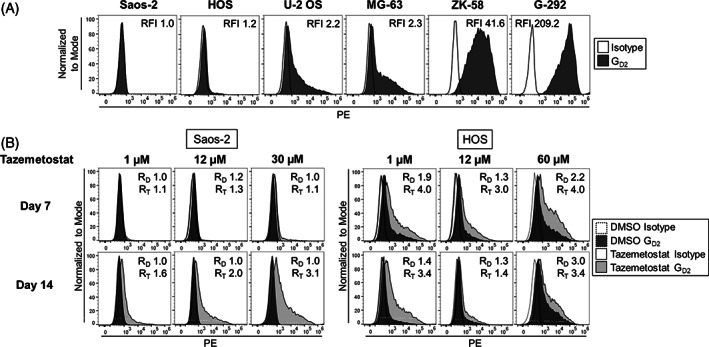
Pretreatment with the EZH2 inhibitor tazemetostat fails to upregulate G_D2_ surface expression in OS cell lines in vitro. A, G_D2_ surface expression in six OS cell lines by flow cytometry. B, G_D2_ surface expression in the G_D2_‐negative OS cell lines Saos‐2 and HOS by flow cytometry on days 7 and 14 of treatment with 1 μM, 12 μM, and 30 μM (Saos‐2) or 60 μM (HOS) tazemetostat, respectively. Equivalent volumes of DMSO were used as controls. For 12 μM, representative experiments of three are shown. R_D/T_=RFI after incubation with DMSO/tazemetostat

Analysis of G_D2_ surface expression in 6 OS cell lines by flow cytometry identified four OS cell lines with G_D2_ surface expression at variable levels and two G_D2_‐negative cell lines (defined as RFI<2), Saos‐2 and HOS (Figure 1A). To investigate whether EZH2 inhibition induces G_D2_ expression in OS, as previously described in Ewing sarcoma,^16^ we incubated Saos‐2 and HOS cells with increasing concentrations of the EZH2 inhibitor tazemetostat and analyzed G_D2_ surface expression on days 7 and 14. Tazemetostat up to concentrations of 12 μM failed to consistently increase G_D2_ expression in the two cell lines (Figure 1B). Prior to further dose escalation, we quantified the viability of the tumor cells in the presence of tazemetostat at concentrations up to 100 μM in a 3‐day in vitro assay. At 30 μM and 60 μM tazemetostat, respectively, approximately 70% of Saos‐2 and HOS cells remained viable (30% inhibitory concentration, IC_30_; Figure S1). Even at the individual IC_30_ values, tazemetostat only slightly enhanced G_D2_ expression in these OS cell lines within 14 days (Figure 1B). In conclusion, in contrast to Ewing sarcoma, pretreatment with EZH2 inhibitors is not effective to overcome low and heterogeneous G_D2_ surface expression in OS.

### 3.2 G_D2_ expression in OS depends on cell confluency

**FIGURE 2:**
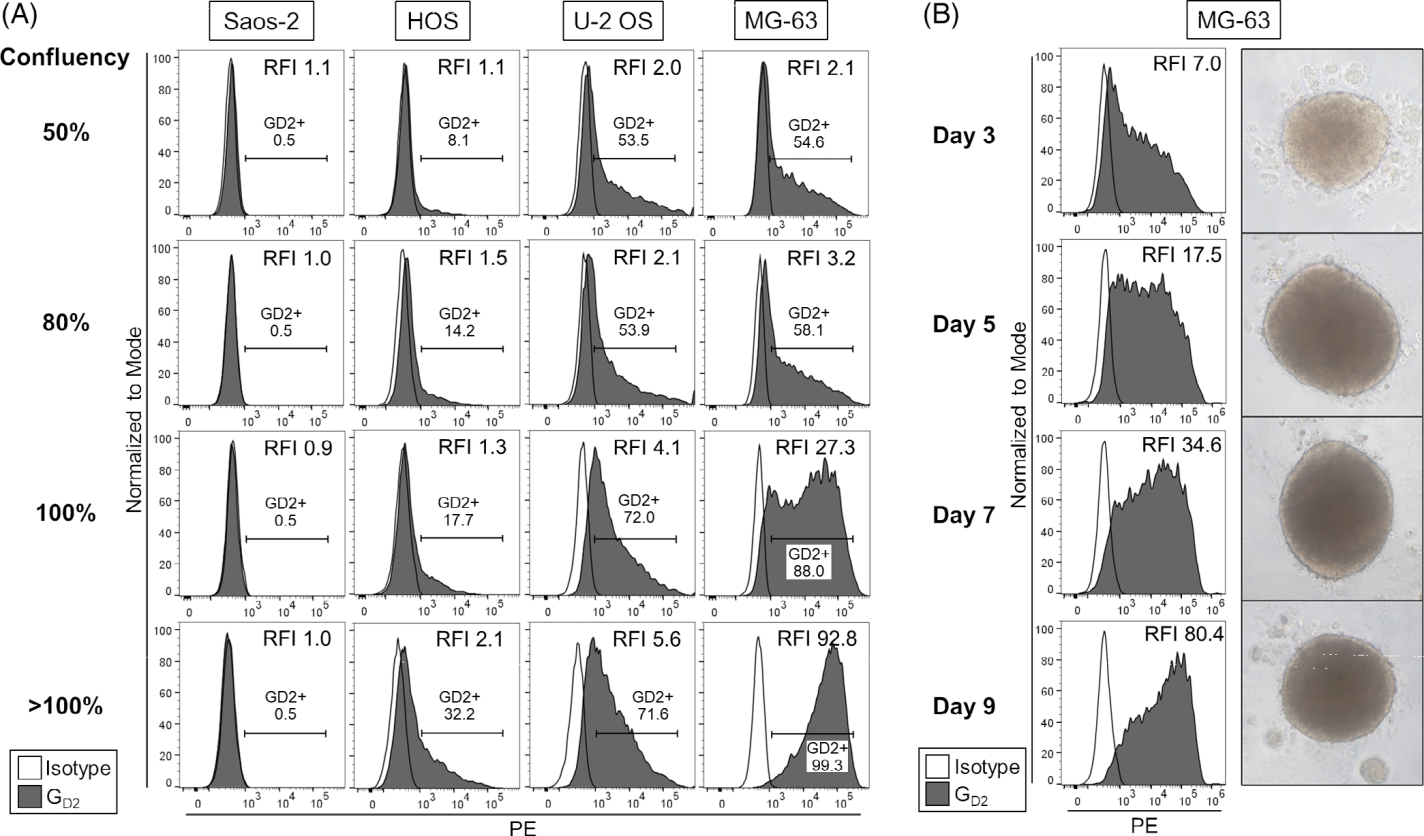
G_D2_ surface expression among and within individual OS cell lines is variable and increases with cell confluency. A, G_D2_ surface expression in four OS cell lines at different stages of in vitro cell confluency by flow cytometry. Representative experiments of two are shown. B, G_D2_ surface expression by flow cytometry on MG‐63 cells derived from growing tumor spheroids over a 9‐day culture period under anchorage‐independent culture conditions. Shown are histograms and photographs (100‐fold level of magnification) of one representative experiment of two

While screening OS cell lines for G_D2_ expression, we noticed that expression levels vary within individual cell lines during cell culture. To test the hypothesis that G_D2_ expression levels vary with cell confluencies, we performed a systematic analysis of G_D2_ surface expression by flow cytometry in four OS cell lines at different stages of confluency (50%, 80%, 100%, >100%). Whereas the Saos‐2 cell line remained G_D2_neg (RFI_50%_: median 1.0, range 0.8‐1.1; RFI_>100%_: median 1.0, range 1.0‐1.0) throughout in vitro cell culture, anti‐G_D2_ fluorescence intensities substantially increased with cell confluency in MG‐63 (RFI_50%_: median 1.8, range 1.5‐2.1; RFI_>100%_: median 103.5, range 92.8‐114.2) and to a lesser degree in U‐2 OS (RFI_50%_: median 1.9, range 1.7‐2.0; RFI_>100%_: median 4.5, range 3.4‐5.6) and in HOS (RFI_50%_: median 1.0, range 0.9‐1.1; RFI_>100%_: median 2.2, range 2.1‐2.2) (Figure 2A). In parallel, the percentages of OS cells expressing G_D2_ above background, defined by the isotype control, increased from a median of 48.9% (range_50%_ 43.1%‐54.6%) to 99.5% (range_>100%_ 99.3%‐99.7%) in MG‐63, from 7.3% (range_50%_ 6.5%‐8.1%) at 50% confluency to a median of 36.3% (range_>100%_ 32.3%‐40.4%) in postconfluent HOS cells and from 49.9% (range_50%_ 46.3%‐53.5%) to 66.8% (range_>100%_ 59.0%‐74.5%) in U‐2 OS (Figure 2A). Overall, G_D2_ surface expression increased with higher cell confluencies in three of four OS cell lines.

To investigate whether confluency‐dependent upregulation of G_D2_ in OS can be reproduced in three‐dimensional structures, we assessed G_D2_ surface expression by flow cytometry on days 3, 5, 7, and 9 of multicellular spheroid culture of MG‐63 cells. Indeed, G_D2_ expression levels noticeably increased during spheroid growth (RFI_d3_: median 5.5, range 3.9‐7.0; RFI_d5_: median 18.1, range 17.5‐18.6; RFI_d7_: median 35.2, range 34.6‐35.8; RFI_d9_: 80.4) (Figure 2B). Thus, cell density is associated with G_D2_ surface expression not only in monolayer cultures, but also in three‐dimensional tumor spheroids mimicking micrometastatic tumor growth.

### 3.3 Confluency‐dependent upregulation of G_D2_ in OS cells depends on an intact Golgi apparatus

**FIGURE 3:**
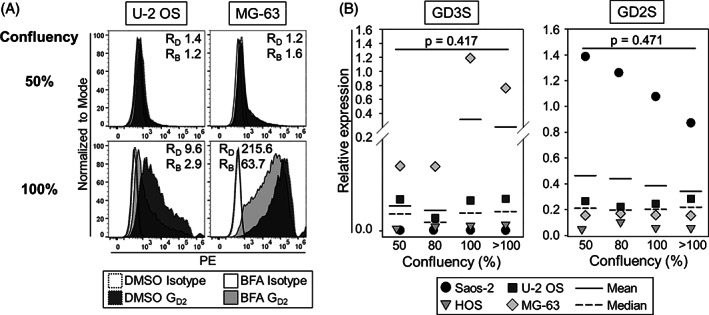
The influence of cell confluency on G_D2_ biosynthesis in OS. A, G_D2_ surface expression by flow cytometry on the OS cell lines U‐2 OS and MG‐63 after addition of 5 mg/mL brefeldin A (BFA) or DMSO (control) to the cell cultures 24 hours before reaching 50% or 100% confluency, respectively. R_D/B_=RFI after incubation with DMSO/BFA. B, GD3S and GD2S gene expression in four OS cell lines at different stages of confluency by qRT‐PCR. Relative expression was determined by calculating Delta Ct with a reference gene. Experiments were repeated twice. Statistical analysis was performed with one‐way repeated measures ANOVA Shown

Subsequent experiments aimed at understanding mechanisms of the observed dynamic, confluency‐dependent regulation of G_D2_ expression in OS. Ganglioside *de novo* biosynthesis depends on the activity of glycosyltransferases located in the Golgi. To understand the contribution of *de novo* synthesis to G_D2_ upregulation in OS, we used BFA^22^ to inhibit Golgi apparatus function in the OS cell lines U‐2 OS and MG‐63 at 50% and 100% confluency, respectively, followed by analysis of G_D2_ surface expression. Whereas basic low‐level expression of G_D2_ at low confluencies was not affected, BFA noticeably counteracted upregulation of G_D2_ in confluent cells (Figure 3A). We conclude that additional surface G_D2_ in confluent OS cells originates from the Golgi as a product of *de novo* synthesis. To understand the mechanism in more detail, we next studied individual steps along the synthetic pathway of G_D2_ in OS for confluency‐dependent effects.

### 3.4 Cell confluency can be associated with increased GD3 synthase expression in OS cell lines

Ganglioside biosynthesis is regulated on a transcriptional level by differential expression of glycosyltransferase genes. To investigate whether transcriptional activity of the genes encoding for the two critical enzymes in G_D2_ synthesis, GD3S or GD2S,^23^ underlies cell confluency‐dependent dynamics, we quantified their expression at different stages of confluency in the four OS cell lines by qRT‐PCR (Figure 3B). Median relative expression of the GD2S gene remained constant in all four OS cell lines, irrespective of changes in confluency (50% median 0.21, range 0.05‐1.39, >100% median 0.22, range 0.05‐0.90). Median relative expression of GD3S gene was not affected by confluency in three of the four cell lines (50% median: 0.001, range 0.001‐0.1, 100% median: 0.001, range 0.001‐0.76), but increased in the cell line MG‐63 after reaching confluency (50%: 0.1, 100% 1.2), concomitant with strong upregulation of G_D2_ surface expression (Figure 2A). We conclude that confluency‐dependent upregulation of G_D2_ expression can be associated with GD3S gene expression at least in individual OS cell lines.

### 3.5 GD3S 5′UTR does not regulate GD3S mRNA translation in a cell confluency‐dependent manner in MG‐63 OS cells

GD3S gene expression in OS could also be affected by translational regulation. The predicted structure of the 5′ untranslated region (5′UTR) of the GD3S mRNA suggests the presence of internal ribosomal entry sites (IRES, Figure S2A^24^) which can act as barriers to conventional cap‐dependent ribosomal scanning.^19^ Binding of IRES trans‐acting factors (ITAF) to the 5′UTR can initiate translation in a cap‐independent way (Figure S2B). To investigate whether the GD3S 5′UTR in OS differentially regulates translation dependent on cell confluency, we transfected MG‐63 OS cells with a bicistronic vector containing the reporter gene GFP downstream of the GD3S 5′UTR (pCAT‐5′UTR‐GFP) to indicate 5′‐driven translational activity, or with pCAT‐GFP as control, then analyzed GFP expression at 50% and 100% confluency by flow cytometry. Insertion of the GD3S 5′UTR indeed noticeably decreases GFP expression, but the translational block imposed by the 5′UTR is not relieved by cell confluency (Figure S2C). Thus, the GD3S 5′UTR does not contribute to confluency‐dependent dynamics of GD3S and therewith G_D2_ expression in MG‐63 OS cells.

### 3.6 PKC stimulation does not affect G_D2_ expression in OS cells

The dynamic expression of G_D2_ in OS cells suggests a fast‐acting regulatory mechanism. One candidate is activation of the PKC, which can accelerate vesicular transport of antigens from the Golgi to the plasma membrane, thereby increasing antigen surface expression.^25^ Studies in the neuroblastoma/glioma hybrid cell line NG108‐15 have found that PKC stimulation of low‐confluent cells induces characteristic ganglioside expression patterns, mimicking those of fully confluent cells.^26^ To investigate whether PKC is involved in regulation of G_D2_ surface expression in OS, we stimulated low‐confluent U‐2 OS and MG‐63 cells with the PKC modulators PMA (Figure S3A) or bryostatin 1 (Figure S3B), followed by analysis of G_D2_ surface expression using flow cytometry. G_D2_ expression levels remained low and unchanged by PKC‐stimulation. Thus, PKC activation cannot mimic confluency‐dependent upregulation of G_D2_ in OS cells.

### 3.7 Confluency affects in vitro cytolysis of OS cells by G_D2_ ‐specific CAR T cells

**FIGURE 4:**
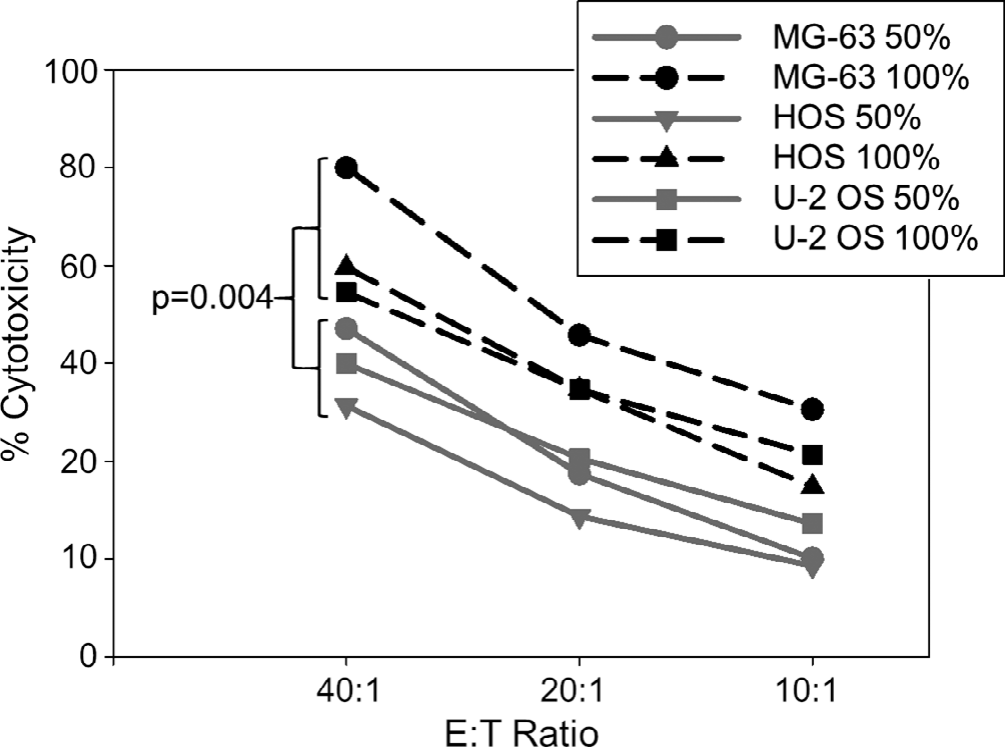
In vitro cytolysis of OS cells by G_D2_‐specific CAR T cells depends on cell confluency. Cytolysis of HOS, U‐2 OS, and MG‐63 cells at 50% or 100% confluency after 4 hours of coincubation with GD2‐BBζ‐transduced T cells. Statistical analysis by paired *t*‐test for all E:T ratios

Toward our goal of using G_D2_ as an immunotherapeutic target antigen in OS, we investigated whether confluency‐dependent variation in G_D2_ expression affects in vitro cytolysis of OS cells by G_D2_‐redirected T cells. Human T cells were gene‐modified to express the G_D2_‐specific CAR GD2‐BBζ^27^ and co‐cultured with HOS, U‐2 OS, and MG‐63 target cells at 50% or 100% confluencies, respectively. High cell confluencies significantly enhanced CAR T‐cell‐mediated in vitro cytolysis of all OS cell targets in a cytotoxicity assay (Figure 4). Thus, high cell confluencies associated with increased G_D2_ target expression enhance the sensitivity of OS to G_D2_‐specific CAR T‐cell therapy.

## 4 DISCUSSION

Due to its restricted tissue expression, the disialoganglioside G_D2_ is an attractive target for cancer immunotherapy.^28^ G_D2_‐specific monoclonal antibodies are approved for the treatment of high‐risk neuroblastoma,^10^, ^29^ a cancer with abundant and consistent G_D2_ expression. In addition, G_D2_‐specific CAR T cells are starting to show clinical potential in this cancer.^9^ Extending the impact of G_D2_‐targeted therapies beyond neuroblastoma is challenged by low levels and heterogeneity of G_D2_ expression in other malignancies. Consistent with the literature,^12^, ^15^, ^30^, ^31^ we found G_D2_ surface expression in the majority of OS cells lines, but levels were highly variable.

We previously reported evidence that biosynthesis of G_D2_ in Ewing sarcoma underlies epigenetic regulation involving EZH2, the catalytic component of the Polycomb Repressor Complex 2 (PCR2).^16^ Our new finding that EZH2 is not a major regulator of G_D2_ expression in OS was not unexpected: Whereas Ewing sarcoma is driven by a disease‐defining translocation, with consistent high‐level EZH2 expression as a direct consequence of the resulting fusion protein,^32^ OS is characterized by a disorganized genome with highly variable and complex chromosomal alterations.^33^ Even though EZH2 can be overexpressed in OS,^17^, ^18^ loss‐of‐function of PRC2 was reported in OS cell lines, including HOS and U‐2 OS.^17^ Thus, using EZH2 inhibitors for sensitizing cancer cells to G_D2_‐targeted therapy may be a valuable option in Ewing sarcoma, but not in OS. Our attempts at pharmacologic upregulation of G_D2_ in OS by the use of PKC modulators, effective to enhance expression of CD22 in B‐cell malignancies^25^, ^34^ and gangliosides in neuroblastoma,^26^ were also unsuccessful.

We observed that G_D2_ in OS varies not only among, but also within individual OS cell lines, and that expression levels correlate with cell confluency, both in monolayer cultures and in tumor spheroids. Moreover, we found that confluency‐dependent upregulation of G_D2_ in OS cells is mediated by increased *de novo* synthesis in the Golgi apparatus. We further show that cell confluency can induce expression of the key enzyme in G_D2_ biosynthesis, GD3S, whereas regulatory elements in the 5′UTR of the GD3S gene or activation of PKC are not affected. We still have to unravel the detailed mechanisms by which higher confluency in OS cells induces expression of GD3S and ultimately G_D2_. Moreover, since a clear association between cell confluency and GD3S expression was shown only in one of four cell lines, additional and alternative mechanisms how cell confluency affects G_D2_ surface expression, for example, by enhancing transport to the cell surface or reducing degradation to less complex gangliosides, must also be considered.

G_D2_ upregulation could be part of a cell response to metabolic stress caused by limited availability of nutrients and oxygen in confluent cell cultures and growing tumors.^35^ Indeed, microenvironmental stress factors, such as hypoxia, low nutrient availability or drug exposure, can induce epigenetic remodeling associated with extensive phenotypic changes.^36^, ^37^ More specifically, cellular hypoxia can induce expression of GD3S^38^ and also Sialin,^39^ a sialic acid transporter, thereby enhancing expression of sialogangliosides in tumor cells. Finally, oxidative stress caused by nutrient deprivation was found to induce expression of G_D2_ in breast cancer cells,^40^, ^41^ concomitant with a cancer stem cell‐like phenotype.^42^ Overall, it is a common observation that ganglioside expression patterns in tumor cells vary with environmental conditions, as mimicked in vitro by cellular confluency. Translating confluency‐related target upregulation into an in vivo strategy is likely to be challenging due to its multifactorial origin, limiting its clinical potential.

To what extent cell density‐dependent regulation of G_D2_ will limit the efficacy of G_D2_‐targeted immunotherapy, for example, by escape of single disseminated tumor cells, remains speculative. Only clinical studies can assess the potential of G_D2_ as a target antigen in OS. Anti‐G_D2_ antibody was found to have no significant efficacy against OS in a phase II trial.^43^ Several G_D2_‐specific CAR T‐cell trials that include OS patients are ongoing (listed in Reference 44). By demonstrating that optimal G_D2_‐CAR T‐cell‐mediated cytotoxicity in OS depends on cell confluency, our data support the need of strategies for overcoming heterogeneous expression of this target. An alternative means to counteract resistance of tumor cells with low antigen expression is to lower the threshold for CAR T‐cell signaling and activation by modulating inherent signaling domains.^45^ Tuning the reactivity of G_D2_‐specific CAR T cells must be weighed against potential on‐target toxicities, for example, on neuronal cells with low‐level G_D2_ expression, and against a risk for tonic T‐cell stimulation triggering rapid exhaustion.^46^ Dual or even triple antigen targeting could be a promising strategy to allow eradication of heterogeneous subpopulations within OS. Besides the carbohydrate G_D2_, the proteins B7H3 and HER2 are candidate antigens for immunotherapeutic targeting of OS.^47^, ^48^ A phase I/II clinical study of second generation HER2‐CAR T cells in OS has demonstrated safety and first evidence of activity.^47^ B7H3‐specific CAR T cells have shown significant in vivo activity against xenograft models of OS and other pediatric tumors,^48^ and are now entering clinical trials.

We conclude that cell confluency‐associated factors by a yet unknown mechanism affect G_D2_ expression and sensitivity to G_D2_‐specific CAR T‐cell targeting in OS cell lines. Combinatorial strategies, such as pretreatment with agents that specifically upregulate the target antigen or multispecific CARs, remain an area of important research to increase the impact of cellular immunotherapy in the management of OS.

## CONFLICT OF INTEREST

The authors do not have any conflict of interests to declare.

## ETHICAL STATEMENT

The use of blood samples from healthy donors was approved by the institutional Ethical Board (Ethik‐Kommission der Ärztekammer Westfalen‐Lippe und der Westfälischen Wilhelms‐Universität Münster, Reference 1IXRös1).

## AUTHORS' CONTRIBUTIONS

All authors had full access to the data in the study and take responsibility for the integrity of the data and the accuracy of the data analysis. *Conceptualization*, S.K., B.A., C.R.; *Methodology*, S.K., B.A., K.G., S.K.; *Investigation*, M.W., S.K., B.A., C.R.; *Formal Analysis*, M.W., S.K., B.A., J.M., K.G., S.K., C.R.; *Resources*, R.C.; *Writing ‐ Original Draft*, M.W., S.K., B.A., C.R.; *Writing ‐ Review & Editing*, M.W., S.K., B.A., J.M., K.G., S.K., C.R.; *Visualization*, M.W., S.K., B.A., C.R.; *Supervision*, S.K., B.A., K.G., C.R.; *Funding Acquisition*, R.C.; *Data Curation*, M.W., J.M., K.G., S.K.; *Validation*, M.W., S.K., B.A., C.R.; *Project Administration*, R.C.

## Supporting information


**FIGURE S1.** OS shows different levels of sensitivity to tazemetostat. Viability of OS cell lines Saos‐2 and HOS following 3‐day incubation with different concentrations of tazemetostat using the CellTiter Glo Luminescent Cell Viability Assay (Promega, Germany). IC_30_=30% inhibitory concentration.

**FIGURE S2.** The GD3S 5′UTR does not regulate confluency‐dependent changes in GD3S expression in MG‐63 OS cells. (A) Secondary structure of the 5′UTR of the GD3S‐mRNA as calculated by the Mfold web server for nucleic acid folding and hybridization prediction. (B) Schematic illustration of IRES‐mediated, cap‐independent translation initiation. IRES = internal ribosomal entry site, ITAF = IRES trans‐acting factors. (C) GFP expression by flow cytometry in MG‐63 cells transfected with pCAT‐GFP or pCAT‐5′UTR‐GFP plasmid at 50% versus 100% confluency.

**FIGURE S3.** Regulation of G_D2_ expression in OS does not involve PKC stimulation. G_D2_ expression by flow cytometry in OS cell lines U‐2 OS and MG‐63 following incubation with (A) 200 nM PMA for 2‐12 hours or (B) 1 ng/mL bryostatin 1 for 24 to 72 hours at low confluencies. Equal amounts of DMSO were used as controls. R_D/P/B_=RFI after incubation with DMSO/PMA/bryostatin 1.

## Data Availability

The data that support the findings of this study are available from the corresponding author upon reasonable request.

## References

[1] Link MP , Allen GM , Horowitz M , et al. Adjuvant chemotherapy of high‐grade osteosarcoma of the extremity: updated results of the multi‐institutional osteosarcoma study. Clin Orthop Relat Res. 1991;270:8‐14. 1884563

[2] Marina NM , Smeland S , Bielack SS , et al. Comparison of MAPIE versus MAP in patients with a poor response to preoperative chemotherapy for newly diagnosed high‐grade osteosarcoma (EURAMOS‐1): an open‐label, international, randomised controlled trial. Lancet Oncol. 2016;17(10):1396‐1408. 10.1016/S1470-2045(16)30214-5. 27569442 PMC5052459

[3] Meyers PA , Schwartz CL , Krailo MD , et al. Osteosarcoma: the addition of muramyl tripeptide to chemotherapy improves overall survival ‐a report from the Children's Oncology Group. J Clin Oncol. 2008;26(4):633‐638. 10.1200/JCO.2008.14.0095. 18235123

[4] Chou AJ , Kleinerman ES , Krailo MD , et al. Addition of muramyl tripeptide to chemotherapy for patients with newly diagnosed metastatic osteosarcoma: a report from the Children's Oncology Group. Cancer. 2009;115(22):5339‐5348. 10.1002/cncr.24566. 19637348 PMC2783515

[5] Bielack SS , Kempf‐Bielack B , Branscheid D , et al. Second and subsequent recurrences of osteosarcoma: presentation, treatment, and outcomes of 249 consecutive cooperative osteosarcoma study group patients. J Clin Oncol. 2009;27(4):557‐565. 10.1200/JCO.2008.16.2305. 19075282

[6] Locke FL , Ghobadi A , Jacobson CA , et al. Long‐term safety and activity of axicabtagene ciloleucel in refractory large B‐cell lymphoma (ZUMA‐1): a single‐arm, multicentre, phase 1–2 trial. Lancet Oncol. 2019;20(1):31‐42. 10.1016/S1470-2045(18)30864-7. 30518502 PMC6733402

[7] Maude SL , Laetsch TW , Buechner J , et al. Tisagenlecleucel in children and young Adults with B‐Cell Lymphoblastic Leukemia. N Engl J Med. 2018;378(5):439‐448. 10.1056/NEJMoa1709866. 29385370 PMC5996391

[8] Rossig C , Kailayangiri S , Jamitzky S , Altvater B . Carbohydrate targets for CAR T cells in solid childhood cancers. Front Oncol. 2018;8:513. 10.3389/fonc.2018.00513. 30483473 PMC6240699

[9] Straathof K , Flutter B , Wallace R , et al. Antitumor activity without on‐target off‐tumor toxicity of GD2–chimeric antigen receptor T cells in patients with neuroblastoma. Sci Transl Med. 2020;12(571): eabd6169. 10.1126/scitranslmed.abd6169. 33239386

[10] Yu AL , Gilman AL , Ozkaynak MF , et al. Anti‐GD2 antibody with GM‐CSF, interleukin‐2, and isotretinoin for neuroblastoma. N Engl J Med. 2010;363(14):1324–1334. 10.1056/NEJMoa0911123. 20879881 PMC3086629

[11] Kailayangiri S , Altvater B , Meltzer J , et al. The ganglioside antigen G(D2) is surface‐expressed in Ewing sarcoma and allows for MHC‐independent immune targeting. Br J Cancer. 2012;106(6):1123‐1133. 10.1038/bjc.2012.57. 22374462 PMC3304425

[12] Roth M , Linkowski M , Tarim J , et al. Ganglioside GD2 as a therapeutic target for antibody‐mediated therapy in patients with osteosarcoma. Cancer. 2014;120(4):548‐554. 10.1002/cncr.28461. 24166473 PMC3946333

[13] Dobrenkov K , Ostrovnaya I , Gu J , Cheung IY , Cheung N‐KV . Oncotargets GD2 and GD3 are highly expressed in sarcomas of children, adolescents, and young adults. Pediatr Blood Cancer. 2016;63(10):1780‐1785. 10.1002/pbc.26097. 27304202 PMC5215083

[14] Long AH , Highfill SL , Cui Y , et al. Reduction of MDSCs with all‐trans retinoic acid improves CAR therapy efficacy for sarcomas. Cancer Immunol Res. 2016;4(10):869‐880. 10.1158/2326-6066.CIR-15-0230. 27549124 PMC5050151

[15] Poon VI , Roth M , Piperdi S , et al. Ganglioside GD2 expression is maintained upon recurrence in patients with osteosarcoma. Clin Sarcoma Res. 2015;5(1):4. 10.1186/s13569-014-0020-9. 25642322 PMC4311500

[16] Kailayangiri S , Altvater B , Lesch S , et al. EZH2 inhibition in Ewing sarcoma upregulates GD2 expression for targeting with gene‐modified T cells. Mol Ther. 2019;27(5):933‐946. 10.1016/j.ymthe.2019.02.014. 30879952 PMC6520468

[17] Feng H , Tillman H , Wu G , Davidoff AM , Yang J . Frequent epigenetic alterations in polycomb repressive complex 2 in osteosarcoma cell lines. Oncotarget. 2018;9(43):27087‐27091. 10.18632/oncotarget.25484. 29930752 PMC6007463

[18] Sun R , Shen J , Gao Y , et al. Overexpression of EZH2 is associated with the poor prognosis in osteosarcoma and function analysis indicates a therapeutic potential. Oncotarget. 2016;7(25):38333–38346. 10.18632/oncotarget.9518. 27223261 PMC5122393

[19] Grobe K , Esko JD . Regulated translation of heparan sulfate N‐acetylglucosamine N‐deacetylase/n‐sulfotransferase isozymes by structured 5′‐untranslated regions and internal ribosome entry sites. J Biol Chem. 2002;277(34):30699‐30706. 10.1074/jbc.M111904200. 12070138

[20] Rossig C , Bollard CM , Nuchtern JG , Merchant DA , Brenner MK . Targeting of G(D2)‐positive tumor cells by human T lymphocytes engineered to express chimeric T‐cell receptor genes. Int J Cancer. 2001;94(2):228‐236. 10.1002/ijc.1457. 11668503

[21] Altvater B , Landmeier S , Pscherer S , et al. 2B4 (CD244) signaling by recombinant antigen‐specific chimeric receptors costimulates natural killer cell activation to leukemia and neuroblastoma cells. Clin Cancer Res. 2009;15(15):4857‐4866. 10.1158/1078-0432.CCR-08-2810. 19638467 PMC2771629

[22] Nebenführ A , Ritzenthaler C , Robinson DG . Brefeldin A: deciphering an enigmatic inhibitor of secretion. Plant Physiol. 2002;130(3):1102‐1108. 10.1104/pp.011569. 12427977 PMC1540261

[23] Ngamukote S , Yanagisawa M , Ariga T , Ando S , Yu RK . Developmental changes of glycosphingolipids and expression of glycogenes in mouse brains. J Neurochem. 2007;103(6):2327‐2341. 10.1111/j.1471-4159.2007.04910.x. 17883393

[24] Zuker M . Mfold web server for nucleic acid folding and hybridization prediction. Nucleic Acids Res. 2003;31(13):3406‐3415. 10.1093/nar/gkg595. 12824337 PMC169194

[25] Biberacher V , Decker T , Oelsner M , et al. The cytotoxicity of anti‐CD22 immunotoxin is enhanced by bryostatin 1 in B‐cell lymphomas through CD22 upregulation and PKC‐βII depletion. Haematologica. 2012;97(5):771‐779. 10.3324/haematol.2011.049155. 22180432 PMC3342982

[26] Bieberich E , Freischütz B , Liour S‐S , Yu RK . Regulation of ganglioside metabolism by phosphorylation and dephosphorylation. J Neurochem. 1998;71(3):972‐979. 10.1046/j.1471-4159.1998.71030972.x. 9721722

[27] Kailayangiri S , Altvater B , Spurny C , et al. Targeting Ewing sarcoma with activated and GD2‐specific chimeric antigen receptor‐engineered human NK cells induces upregulation of immune‐inhibitory HLA‐G. Oncoimmunology. 2017;6(1):e1250050. 10.1080/2162402X.2016.1250050. 28197367 PMC5283645

[28] Cheever MA , Allison JP , Ferris AS , et al. The prioritization of cancer antigens: a national cancer institute pilot project for the acceleration of translational research. Clin Cancer Res. 2009;15(17):816–828. 10.1158/1078-0432.CCR-09-0737. PMC5779623 19723653

[29] Kushner BH , Cheung IY , Modak S , Basu EM , Roberts SS , Cheung NK . Humanized 3F8 anti‐GD2 monoclonal antibody dosing With granulocyte‐macrophage colony‐stimulating factor in patients with resistant neuroblastoma: a phase 1 clinical trial. JAMA Oncol. 2018;4(12):1729–1735. 10.1001/jamaoncol.2018.4005. 30326045 PMC6440722

[30] Dobrenkov K , Cheung N‐KV . GD2‐targeted immunotherapy and radioimmunotherapy. Semin Oncol. 2014;41(5):589‐612. 10.1053/j.seminoncol.2014.07.003. 25440605 PMC4254523

[31] Shibuya H , Hamamura K , Hotta H , et al. Enhancement of malignant properties of human osteosarcoma cells with disialyl gangliosides GD2/GD3. Cancer Sci. 2012;103(9):1656‐1664. 10.1111/j.1349-7006.2012.02344.x. 22632091 PMC7659376

[32] Richter GH , Plehm S , Fasan A , et al. EZH2 is a mediator of EWS/FLI1 driven tumor growth and metastasis blocking endothelial and neuro‐ectodermal differentiation. Proc Natl Acad Sci U S A. 2009;106(13):5324‐5329. 10.1073/pnas.0810759106. 19289832 PMC2656557

[33] Smida J , Xu H , Zhang Y , et al. Genome‐wide analysis of somatic copy number alterations and chromosomal breakages in osteosarcoma. Int J Cancer. 2017;141(4):816–828. 10.1002/ijc.30778. 28494505

[34] Ramakrishna S , Highfill SL , Walsh Z , et al. Modulation of target antigen density improves CAR T‐cell functionality and persistence. Clin Cancer Res. 2019;25(17):5329‐5341. 10.1158/1078-0432.CCR-18-3784. 31110075 PMC8290499

[35] Sheta EA , Trout H , Gildea JJ , Harding MA , Theodorescu D . Cell density mediated pericellular hypoxia leads to induction of HIF‐1α via nitric oxide and Ras/MAP kinase mediated signaling pathways. Oncogene. 2001;20(52):7624‐7634. 10.1038/sj.onc.1204972. 11753640

[36] Al Emran A , Marzese DM , Menon DR , et al. Distinct histone modifications denote early stress‐induced drug tolerance in cancer. Oncotarget. 2018;9(9):8206‐8222. 10.18632/oncotarget.23654. 29492189 PMC5823586

[37] Ravindran MD , Das S , Krepler C , et al. A stress‐induced early innate response causes multidrug tolerance in melanoma. Oncogene. 2015;34(34):4448‐4459. 10.1038/onc.2014.372. 25417704 PMC4442085

[38] Yin J , Miyazaki K , Shaner RL , Merrill AH , Kannagi R . Altered sphingolipid metabolism induced by tumor hypoxia ‐ new vistas in glycolipid tumor markers. FEBS Lett. 2010;584(9):1872‐1878. 10.1016/j.febslet.2009.11.019. 19913543 PMC2856702

[39] Yin J , Hashimoto A , Izawa M , et al. Hypoxic culture induces expression of sialin, a sialic acid transporter, and cancer‐associated gangliosides containing non‐human sialic acid on human cancer cells. Cancer Res. 2006;66(6):2937‐2945. 10.1158/0008-5472.CAN-05-2615. 16540641

[40] Battula VL , Piyaranthna B , Nguyen K , et al. Abstract P6‐02‐01: metabolic stress induces GD2 expression and cancer stem cell phenotype in triple negative breast cancer. Cancer Res. 2017;77(4 Supplement):P6‐02‐01. 10.1158/1538-7445.SABCS16-P6-02-01. PMC8854435 34811508

[41] Jaggupilli A , Ly SJ , Borkar R , et al. Abstract 3801: oxidative stress induces glutamine‐dependent GD2+ triple negative breast cancer stem cells. Cancer Res. 2020;80(16 Supplement):3801. 10.1158/1538-7445.AM2020-3801.

[42] Battula VL , Shi Y , Evans KW , et al. Ganglioside GD2 identifies breast cancer stem cells and promotes tumorigenesis. J Clin Invest. 2012;122(6):2066‐2078. 10.1172/JCI59735. 22585577 PMC3591166

[43] Hingorani P , Krailo MD , Buxton A , et al. Phase II study of antidisialoganglioside antibody, dinutuximab, in combination with GM‐CSF in patients with recurrent osteosarcoma (AOST1421): a report from the Children's Oncology Group. JCO. 2020;38(15_suppl):10508. 10.1200/JCO.2020.38.15_suppl.10508.

[44] Yoshida K , Okamoto M , Aoki K , Takahashi J , Saito N . A review of T‐cell related therapy for osteosarcoma. Int J Mol Sci. 2020;21(14):4877. 10.3390/ijms21144877. PMC7402310 32664248

[45] Majzner RG , Rietberg SP , Sotillo E , et al. Tuning the antigen density requirement for CAR T‐cell activity. Cancer Discov. 2020;10(5):702‐723. 10.1158/2159-8290.CD-19-0945. 32193224 PMC7939454

[46] Long AH , Haso WM , Shern JF , et al. 4‐1BB costimulation ameliorates T cell exhaustion induced by tonic signaling of chimeric antigen receptors. Nat Med. 2015;21(6):581‐590. 10.1038/nm.3838. 25939063 PMC4458184

[47] Ahmed N , Brawley VS , Hegde M , et al. Human epidermal growth factor receptor 2 (HER2) ‐specific chimeric antigen receptor‐modified T cells for the immunotherapy of HER2‐positive sarcoma. J Clin Oncol. 2015;33(15):1688‐1696. 10.1200/JCO.2014.58.0225. 25800760 PMC4429176

[48] Majzner RG , Theruvath JL , Nellan A , et al. CAR T cells targeting B7‐H3, a pan‐cancer antigen, demonstrate potent preclinical activity against pediatric solid tumors and brain tumors. Clin Cancer Res. 2019;25(8):2560‐2574. 10.1158/1078-0432.CCR-18-0432. 30655315 PMC8456711

