# Supplementary figures and images for: Surface expression of the immunotherapeutic target GD2 in osteosarcoma depends on cell confluency
Source: Cancer Rep (Hoboken). 2021 Apr 2;4(5):e1394. doi: 10.1002/cnr2.1394 (PMC8551999; doi:10.1002/cnr2.1394)

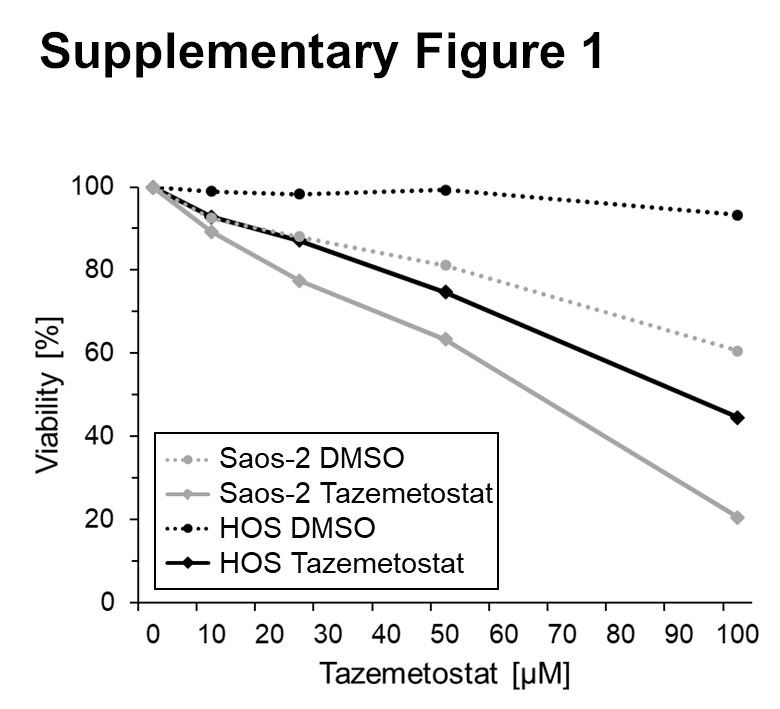

Supplement: Supplementary file 1 — FIGURE S1. OS shows different levels of sensitivity to tazemetostat. Viability of OS cell lines Saos‐2 and HOS following 3‐day incubation with different concentrations of tazemetostat using the CellTiter Glo Luminescent Cell Viability Assay (Promega, Germany). IC30=30% inhibitory concentration. [file CNR2-4-e1394-s003.tif]

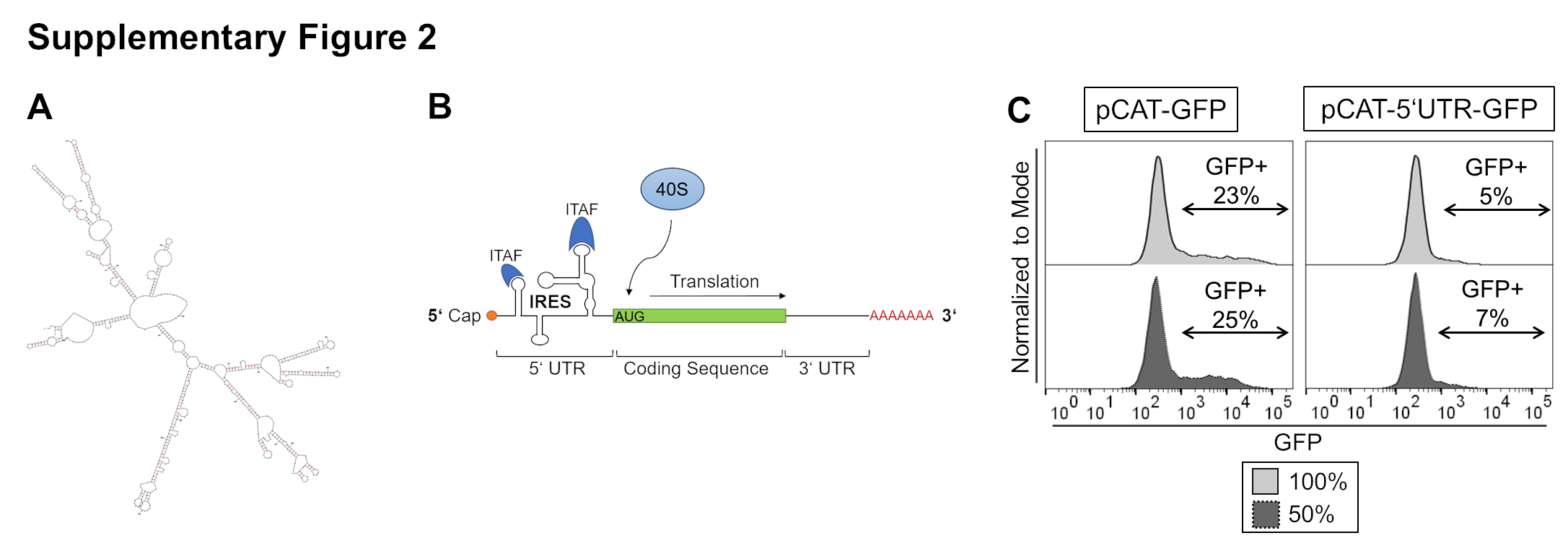

Supplement: Supplementary file 2 — FIGURE S2. The GD3S 5′UTR does not regulate confluency‐dependent changes in GD3S expression in MG‐63 OS cells. (A) Secondary structure of the 5′UTR of the GD3S‐mRNA as calculated by the Mfold web server for nucleic acid folding and hybridization prediction. (B) Schematic illustration of IRES‐mediated, cap‐independent translation initiation. IRES = internal ribosomal entry site, ITAF = IRES trans‐acting factors. (C) GFP expression by flow cytometry in MG‐63 cells transfected with pCAT‐GFP or pCAT‐5′UTR‐GFP plasmid at 50% versus 100% confluency. [file CNR2-4-e1394-s001.tif]

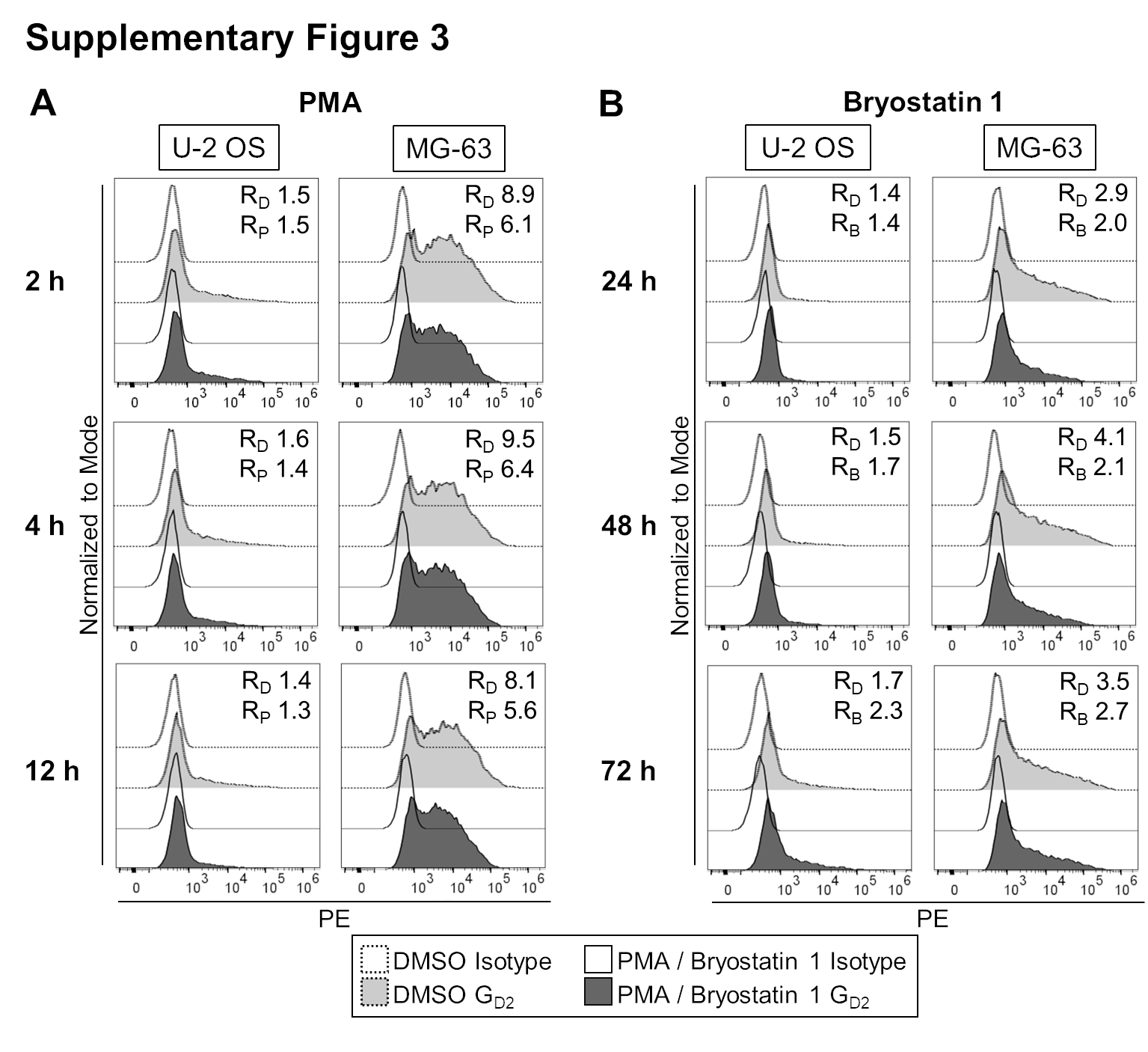

Supplement: Supplementary file 3 — FIGURE S3. Regulation of GD2 expression in OS does not involve PKC stimulation. GD2 expression by flow cytometry in OS cell lines U‐2 OS and MG‐63 following incubation with (A) 200 nM PMA for 2‐12 hours or (B) 1 ng/mL bryostatin 1 for 24 to 72 hours at low confluencies. Equal amounts of DMSO were used as controls. RD/P/B=RFI after incubation with DMSO/PMA/bryostatin 1. [file CNR2-4-e1394-s002.tif]
